# Seasonality of Respiratory Syncytial Virus — United States, 2017–2023

**DOI:** 10.15585/mmwr.mm7214a1

**Published:** 2023-04-07

**Authors:** Sarah Hamid, Amber Winn, Rishika Parikh, Jefferson M. Jones, Meredith McMorrow, Mila M. Prill, Benjamin J. Silk, Heather M. Scobie, Aron J. Hall

**Affiliations:** ^1^Epidemic Intelligence Service, CDC; ^2^Coronavirus and Other Respiratory Viruses Division, National Center for Immunizations and Respiratory Diseases, CDC; ^3^Goldbelt C6, Chesapeake, Virginia.

In the United States, respiratory syncytial virus (RSV) infections cause an estimated 58,000–80,000 hospitalizations among children aged <5 years (*1*,*2*) and 60,000–160,000 hospitalizations among adults aged ≥65 years each year (*3*–*5*). U.S. RSV epidemics typically follow seasonal patterns, peaking in December or January (*6*,*7*), but the COVID-19 pandemic disrupted RSV seasonality during 2020–2022 (*8*). To describe U.S. RSV seasonality during prepandemic and pandemic periods, polymerase chain reaction (PCR) test results reported to the National Respiratory and Enteric Virus Surveillance System (NREVSS)* during July 2017–February 2023 were analyzed. Seasonal RSV epidemics were defined as the weeks during which the percentage of PCR test results that were positive for RSV was ≥3% (*9*). Nationally, prepandemic seasons (2017–2020) began in October, peaked in December, and ended in April. During 2020–21, the typical winter RSV epidemic did not occur. The 2021–22 season began in May, peaked in July, and ended in January. The 2022–23 season started (June) and peaked (November) later than the 2021–22 season, but earlier than prepandemic seasons. In both prepandemic and pandemic periods, epidemics began earlier in Florida and the Southeast and later in regions further north and west. With several RSV prevention products in development,^†^ ongoing monitoring of RSV circulation can guide the timing of RSV immunoprophylaxis and of clinical trials and postlicensure effectiveness studies. Although the timing of the 2022–23 season suggests that seasonal patterns are returning toward those observed in prepandemic years, clinicians should be aware that off-season RSV circulation might continue.

Each week, participating clinical and public health laboratories voluntarily report to NREVSS aggregate numbers of RSV PCR tests performed and numbers of positive test results. Although antigen tests are sometimes performed, this analysis was restricted to PCR tests because they accounted for >90% of tests reported (*9*). Surveillance years were defined based on troughs in RSV circulation. During 2017–2020 (the prepandemic period), surveillance years began in early July (epidemiologic week 27) and ended the following year in late June (week 26). Because the typical winter RSV epidemic was absent during 2020–21, and the 2021–22 epidemic began in the spring, the 2021–22 and 2022–23 surveillance years (pandemic period) were defined as early March (week 9) to late February (week 8) of the following year.^§^ Several methods for characterizing RSV seasonality were explored (Supplementary Table 1, https://stacks.cdc.gov/view/cdc/126381) (Supplementary Table 2, https://stacks.cdc.gov/view/cdc/126380). A 3% test positivity threshold was chosen because it prospectively identified a high proportion of annual RSV detections during epidemic periods of moderate duration. The epidemic onset and offset (or end) weeks were defined, respectively, as the first and last of 2 consecutive weeks when the percentage of PCR tests positive for RSV was ≥3%. The epidemic duration was the inclusive number of weeks between onset and offset. The peak was defined as the week with the highest percentage of PCR tests positive for RSV.

Epidemic onset, offset, peak, and duration were identified for each season at the national level and by U.S. Department of Health and Human Services (HHS) region.^¶^ Because patterns of weekly RSV circulation in Alaska, Florida, and Hawaii are different from those in other states within their assigned regions (HHS Regions 10, 4, and 9, respectively), these states were excluded from regional analyses. State-level seasonality for Florida is reported; however, an insufficient number of laboratories in Alaska and Hawaii consistently reported PCR data to present state-level seasonality in those states. The analysis included data from laboratories that consistently conducted PCR testing.** This activity was conducted consistent with applicable federal law and CDC policy.^††^

During the period with weeks ending July 8, 2017–February 25, 2023, five distinct RSV epidemics occurred: three before the COVID-19 pandemic (2017–18, 2018–19, and 2019–20) and two during the pandemic (2021–22 and 2022–23). Using the 3% epidemic threshold, no seasonal RSV epidemic was observed to occur during the 2020–21 surveillance year (Figure 1). The number of tests performed increased substantially during the pandemic (Table).

**FIGURE 1:**
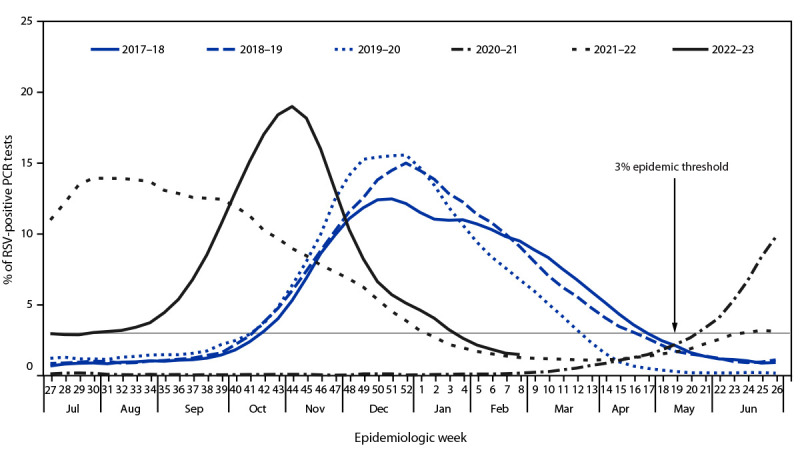
Percentage* of polymerase chain reaction test results positive for respiratory syncytial virus, by epidemiologic week — National Respiratory and Enteric Virus Surveillance System, United States, July 2017–February 2023 **Abbreviations:** PCR = polymerase chain reaction; RSV = respiratory syncytial virus. * Three-week centered moving averages of percentage of RSV-positive PCR test results nationally. The threshold for a seasonal epidemic was set at 3% RSV-positive PCR test results (not based on a moving average).

**TABLE:** Summary of respiratory syncytial virus seasons, by U.S. Department of Health and Human Services Region* and in Florida — National Respiratory and Enteric Virus Surveillance System, July 2017–February 2023^†^

HHS region (headquarters) or state, RSV season	No. of laboratories reporting	No. of tests performed	Onset epidemiologic week^§^ (mo)	Peak epidemiologic week^¶^ (mo)	Offset epidemiologic week** (mo)	Epidemic duration, no. of wks^††^	% of annual detections in epidemic period^§§^
**National**							
**2017–18**	**130**	**810,977**	**42 (Oct) **	**51 (Dec)**	**16 (Apr)**	**27**	**96**
**2018–19**	**138**	**816,512**	**41 (Oct)**	**51 (Dec)**	**16 (Apr)**	**28**	**95**
**2019–20**	**166**	**999,493**	**42 (Oct)**	**51 (Dec)**	**12 (Mar)**	**23**	**95**
**2021–22**	**196**	**1,849,047**	**21 (May)**	**30 (Jul)**	**1 (Jan)**	**33**	**92**
**2022–23**	**221**	**3,160,659**	**24 (Jun)**	**44 (Nov)**	**3 (Jan)**	**32**	**92**
**Region 1 (Boston)**							
2017–18	9	38,902	44 (Nov)	52 (Dec)	17 (Apr)	26	97
2018–19	10	39,951	45 (Nov)	52 (Dec)	15 (Apr)	23	94
2019–20	12	53,441	44 (Nov)	52 (Dec)	12 (Mar)	21	96
2021–22	11	70,122	25 (Jun)	36 (Sep)	51 (Dec)	27	90
2022–23	10	184,128	35 (Sep)	44 (Nov)	50 (Dec)	16	81
**Region 2 (New York City)**							
2017–18	8	52,010	43 (Oct)	1 (Jan)	13 (Mar)	23	93
2018–19	9	62,066	44 (Nov)	51 (Dec)	13 (Mar)	22	89
2019–20	13	100,384	43 (Oct)	49 (Dec)	10 (Mar)	20	90
2021–22	9	186,986	30 (Jul)	39 (Oct)	50 (Dec)	21	78
2022–23	11	286,733	38 (Sep)	45 (Nov)	51 (Dec)	14	74
**Region 3 (Philadelphia)**							
2017–18	11	55,660	42 (Oct)	52 (Dec)	14 (Apr)	25	94
2018–19	9	46,260	43 (Oct)	49 (Dec)	13 (Mar)	23	93
2019–20	13	63,745	43 (Oct)	1 (Jan)	9 (Feb)	19	90
2021–22	16	85,062	24 (Jun)	34 (Aug)	52 (Jan)	29	92
2022–23	13	142,867	23 (Jun)	42 (Oct)	3 (Jan)	33	95
**Region 4 (Atlanta)**							
2017–18	9	55,316	40 (Oct)	51 (Dec)	14 (Apr)	27	92
2018–19	9	59,747	38 (Sep)	52 (Dec)	13 (Mar)	28	92
2019–20	11	60,429	38 (Sep)	48 (Nov)	9 (Feb)	24	92
2021–22	11	130,818	14 (Apr)	30 (Jul)	47 (Nov)	34	86
2022–23	13	267,547	21 (May)	40 (Oct)	50 (Dec)	30	89
**Region 5 (Chicago)**							
2017–18	33	201,222	44 (Nov)	50 (Dec)	17 (Apr)	26	95
2018–19	35	185,950	41 (Oct)	1 (Jan)	12 (Mar)	24	92
2019–20	51	273,402	42 (Oct)	51 (Dec)	11 (Mar)	22	93
2021–22	68	462,017	24 (Jun)	33 (Aug)	49 (Dec)	26	86
2022–23	81	725,015	32 (Aug)	44 (Nov)	2 (Jan)	23	90
**Region 6 (Dallas)**							
2017–18	16	128,254	40 (Oct)	48 (Dec)	17 (Apr)	30	97
2018–19	16	123,577	40 (Oct)	47 (Nov)	13 (Mar)	26	94
2019–20	17	131,460	40 (Oct)	48 (Nov)	11 (Mar)	24	95
2021–22	22	300,954	20 (May)	28 (Jul)	1 (Jan)	34	96
2022–23	21	355,621	17 (Apr)	41 (Oct)	3 (Jan)	39	95
**Region 7 (Kansas City)**							
2017–18	8	24,443	46 (Nov)	7 (Feb)	20 (May)	27	97
2018–19	9	32,138	46 (Nov)	52 (Dec)	18 (May)	25	97
2019–20	9	36,150	43 (Oct)	51 (Dec)	13 (Mar)	23	97
2021–22	14	120,813	21 (May)	33 (Aug)	51 (Dec)	31	91
2022–23	29	247,426	36 (Sep)	44 (Nov)	2 (Jan)	19	88
**Region 8 (Denver)**							
2017–18	9	55,535	48 (Dec)	7 (Feb)	17 (Apr)	22	96
2018–19	9	57,877	48 (Dec)	5 (Feb)	18 (May)	23	97
2019–20	11	64,399	46 (Nov)	4 (Jan)	14 (Apr)	21	97
2021–22	10	119,298	26 (Jul)	39 (Oct)	1 (Jan)	28	92
2022–23	9	115,584	39 (Oct)	45 (Nov)	5 (Feb)	19	90
**Region 9 (San Francisco)**							
2017–18	11	121,569	47 (Nov)	6 (Feb)	16 (Apr)	22	97
2018–19	8	108,118	48 (Dec)	6 (Feb)	17 (Apr)	22	97
2019–20	8	108,085	47 (Nov)	1 (Jan)	13 (Mar)	19	96
2021–22	9	163,200	29 (Jul)	49 (Dec)	6 (Feb)	30	98
2022–23	9	473,657	37 (Sep)	45 (Nov)	4 (Jan)	20	91
**Region 10 (Seattle)**							
2017–18	8	56,212	47 (Nov)	4 (Jan)	15 (Apr)	21	96
2018–19	15	74,851	47 (Nov)	6 (Feb)	17 (Apr)	23	95
2019–20	13	74,837	46 (Nov)	52 (Dec)	12 (Mar)	19	95
2021–22	18	154,248	34 (Aug)	50 (Dec)	5 (Feb)	24	94
2022–23	20	228,081	39 (Oct)	45 (Nov)	5 (Feb)	19	90
**Florida**							
2017–18	6	20,224	32 (Aug)	46 (Nov)	9 (Mar)	30	87
2018–19	7	24,390	29 (Jul)	45 (Nov)	13 (Mar)	37	91
2019–20	5	28,626	33 (Aug)	48 (Nov)	7 (Feb)	27	88
2021–22	5	43,340	12 (Mar)	23 (Jun)	49 (Dec)	38	90
2022–23	2	68,801	18 (May)	40 (Oct)	3 (Jan)	38	90

**Abbreviations:** HHS = U.S. Department of Health and Human Services; PCR = polymerase chain reaction; RSV = respiratory syncytial virus.

* https://www.hhs.gov/about/agencies/iea/regional-offices/index.html. Patterns of weekly RSV circulation in Alaska, Florida, and Hawaii are distinct from other states within their assigned regions (HHS regions 10, 4, and 9, respectively); therefore, these states were excluded from regional analyses. State-level seasonality for Florida is reported; however, there are an insufficient number of laboratories consistently reporting RSV PCR data to present state-level seasonality in Alaska and Hawaii.

^†^ Because the typical seasonal RSV epidemic was notably absent during the 2020–21 surveillance year, data from this surveillance year are not shown. Surveillance years were defined based on troughs in RSV circulation. During 2017–2020, surveillance years began in epidemiologic week 27 (early July) and ended the following year in epidemiologic week 26 (late June). During the COVID-19 pandemic (2021–22 and 2022–23), surveillance years began in epidemiologic week 9 (early March) and ended the following year in epidemiologic week 8 (late February).

^§^ The epidemic onset was defined as the first of 2 consecutive weeks when the percentage of PCR tests positive for RSV was ≥3%.

^¶^ The epidemic peak was defined as the week with the highest percentage of PCR tests positive for RSV.

** The epidemic offset was defined as the last of 2 consecutive weeks when the percentage of PCR tests positive for RSV was ≥3%.

^††^ The epidemic duration was the inclusive number of weeks between onset and offset.

^§§^ Annual percentage of detections in the epidemic period was defined as the proportion of all detections during a surveillance year that occurred during the epidemic period.

Nationally, RSV epidemics during the 3 surveillance years preceding the COVID-19 pandemic (2017–2020) began in October, peaked in December, and lasted a median of 27 weeks before the offset during March–April (Table). In contrast, the 2021–22 epidemic began 21 weeks earlier (May), peaked in July, and lasted 33 weeks until January 2022, although the peak percentage of RSV-positive PCR results (15%) was comparable with that during prepandemic seasons (Figure 1). During the 2022–23 surveillance year, onset occurred in June, the proportion of positive PCR results peaked in November, and the peak was higher (19%) than that during prepandemic seasons (range = 13%–16%). The epidemic lasted 32 weeks until the offset occurred in January. 

In both the prepandemic and pandemic periods, RSV epidemics began earliest in Florida and the Southeast and later in regions further north and west (Figure 2). During the Florida prepandemic seasons, the median onset occurred in August, the peak occurred in November, and the epidemic continued until March (median duration = 30 weeks) (Table) (Supplementary Figure, https://stacks.cdc.gov/view/cdc/126382). In the 10 HHS regions (excluding Alaska, Florida, and Hawaii), the median onset ranged from September in Region 4 to December in Region 8. The median epidemic peaks ranged from November in Region 6 to February in Regions 8 and 9. Median offsets ranged from March in Region 5 to May in Region 7; offsets occurred 2–6 weeks earlier during the 2019–20 surveillance year (i.e., when the COVID-19 pandemic began) compared with the preceding 2 surveillance years. The shortest epidemic periods occurred in Region 10 (median = 21 weeks), and the longest occurred in Region 4 (median = 27 weeks).

**FIGURE 2:**
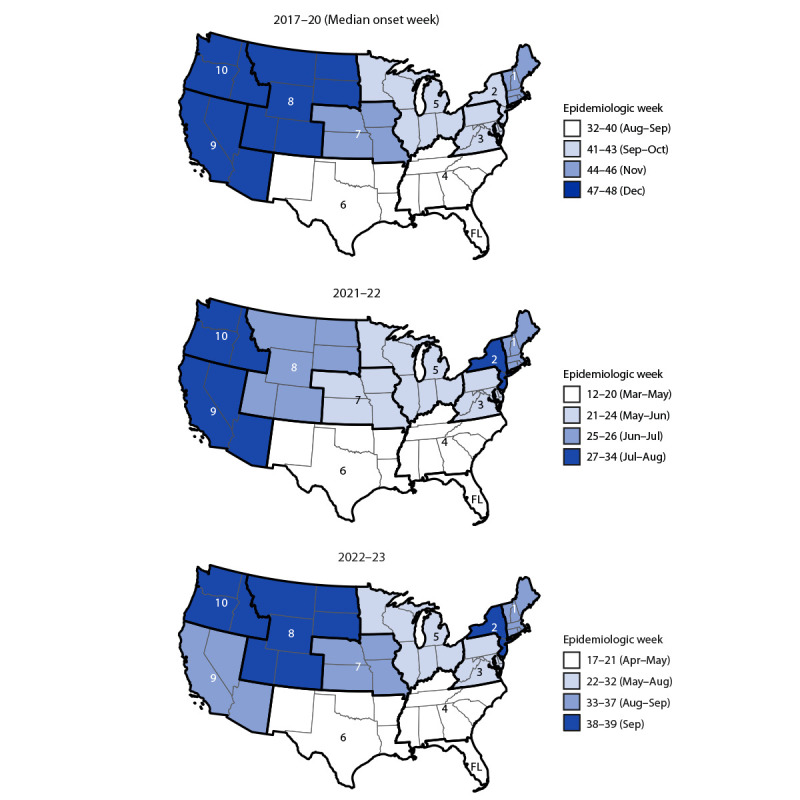
Respiratory syncytial virus epidemic onsets* in U.S. Department of Health and Human Services Regions 1–10^†^ and in Florida — National Respiratory and Enteric Virus Surveillance System, United States, July 2017–February 2023^§^ **Abbreviations**: FL = Florida; RSV = respiratory syncytial virus. * The epidemic onset was defined as the first of 2 consecutive weeks of a surveillance year when the percentage of PCR tests positive for RSV was ≥3%. Median epidemic onset weeks were calculated for the three RSV epidemics that occurred before the COVID-19 pandemic (2017–18, 2018–19, and 2019–20). **^†^** https://www.hhs.gov/about/agencies/iea/regional-offices/index.html. Patterns of weekly RSV circulation in Alaska, Florida, and Hawaii are distinct from other states within their assigned regions; therefore, these states were excluded from regional analyses. State-level seasonality for Florida is reported; however, there are an insufficient number of laboratories consistently reporting polymerase chain reaction testing data to present state-level seasonality in Alaska and Hawaii. **^§^** Surveillance years were defined based on troughs in RSV circulation. During 2017–2020, surveillance years began in epidemiologic week 27 (early July) and ended the following year in epidemiologic week 26 (late June). The aberrant 2020–21 surveillance year was defined as week 27 through week 8 (late February) inclusive. During the COVID-19 pandemic (2021–22 and 2022–23), surveillance years began in epidemiologic week 9 (early March) and ended the following year in epidemiologic week 8.

During the 2021–22 (pandemic) surveillance year, epidemic onsets across the 10 HHS regions and Florida occurred a median of 20 weeks earlier (range = 13–25 weeks) than the median onsets during the prepandemic period (range = March [Florida] to August [Region 10]). Epidemic peaks also occurred earlier than they did during the prepandemic years, ranging from July in Region 6 to December in Region 10. Offsets ranged from November (Region 4) to February (Region 9), which is when prepandemic peaks typically occurred. During the 2021–22 surveillance year, the epidemic durations were a median of 6 weeks longer than the median durations of prepandemic RSV epidemics (range = 21 weeks [Region 2] to 38 weeks [Florida]).

During the 2022–23 season, early epidemic onsets (April–June) were observed in Florida and HHS Regions 3, 4, and 6, but the percentage of RSV-positive PCR test results levelled off before increasing again in September (Figure 1) (Table). In other regions, epidemics began between August and October. Seasons peaked from October in Region 4 to November in regions further north and west (Regions 2, 8, 9, and 10). Epidemics ended between December and February.

## Discussion

In the United States, disruption of the seasonal circulation of RSV was observed during the COVID-19 pandemic as nonpharmaceutical interventions (e.g., school closures and masking) reduced respiratory virus transmission and led to an accumulation of susceptible persons resulting in large epidemics with atypical seasonality (*10*). After the implementation of nonpharmaceutical interventions in March 2020, the 2019–20 RSV epidemic ended earlier than the previous two epidemics. During 2020, RSV circulated at historically low levels. In 2021, RSV circulation began earlier (in late spring), when nonpharmaceutical interventions eased, and continued longer than it did during prepandemic years, although the percentage of RSV-positive PCR tests at the peak was comparable to those during prepandemic years. The 2022–23 epidemic began later than the 2021–22 epidemic but earlier than prepandemic epidemics, suggesting a reversion toward prepandemic seasonality with winter peaks. The peak percentage of positive RSV test results was higher than those in previous years, suggesting higher intensity of circulation. Across both prepandemic and pandemic years, RSV circulation began in Florida and the Southeast and later in regions to the north and west. The consistency of this pattern could help predict the timing of future epidemics in specific regions.

The findings in this report are subject to at least four limitations. First, reporting to NREVSS is voluntary, and analysis is limited to laboratories that consistently report, which might not represent local and state circulation. Second, differences in testing across regions and changes in testing practices and diagnostics over time, including increased panel testing during the COVID-19 pandemic, could have affected the baseline percentage of positive test results and trends, and thus the onset, offset, and duration of epidemics. Third, there is no standard method for characterizing seasonality; seasonal attributes vary depending on the method used. An earlier description of RSV seasonality in the United States used a more sensitive method (retrospective slope 10^§§^) that can only be applied retrospectively and results in longer epidemic durations (*6*,*9*). However, the 3% RSV-positive PCR threshold used in the current analysis can be applied in near real time and identified epidemic periods that included a high concentration of detections (*9*). Finally, this analysis describes regional and national trends; locally available data and region-specific thresholds might better reflect circulation patterns within specific jurisdictions.

Although the peak in RSV circulation during November 2022 suggests that seasonal patterns are returning to those observed in prepandemic years, it is uncertain whether this reversion will continue in the upcoming surveillance year. To monitor RSV circulation, CDC has conducted year-round surveillance using a variety of approaches including active, population-based surveillance for RSV-associated hospitalizations and outpatient visits.^¶¶^ Clinicians should be aware that atypical RSV epidemics might continue and consider testing patients for multiple respiratory pathogens when indicated. With new prevention products nearing licensure, including vaccines for older adults, maternal vaccines, and long-acting RSV immunoprophylaxis for infants and children, policy makers should consider RSV seasonality when making recommendations about the timing of studies and administration of new immunization and other RSV prevention products.

**Summary**

**What is already known about this topic?**

In the United States, the timing of seasonal respiratory syncytial virus (RSV) epidemics (October–April) was disrupted during the COVID-19 pandemic.

**What is added by this report?**

RSV circulation was historically low during 2020–21 and began earlier and continued longer during 2021–22 than during prepandemic seasons. The 2022–23 season started later than the 2021–22 season but earlier than prepandemic seasons, suggesting a return toward prepandemic seasonality.

**What are the implications for public health practice?**

Ongoing monitoring of RSV seasonality can guide the timing of immunoprophylaxis and evaluation of new immunization products. Although an eventual return to prepandemic RSV seasonality is expected, clinicians should be aware that off-season RSV circulation might continue.

## Footnotes

*
https://www.cdc.gov/surveillance/nrevss/index.html

†
https://www.path.org/resources/rsv-vaccine-and-mab-snapshot

§ Defining surveillance year start and end dates based on troughs in RSV activity ensures that seasonal epidemics are encompassed in a 12-month period and that calculated proportions of annual detections that occur in the epidemic period are comparable over time (i.e., that the denominator for the proportion is the 12-month period between troughs in RSV circulation).

¶
https://www.hhs.gov/about/agencies/iea/regional-offices/index.html

** Consistent reporting was defined by the following criteria: 1) reported RSV PCR testing results for ≥30 weeks during the 12-month surveillance year and 2) reported an average of 10 or more RSV-positive PCR tests per week during the surveillance year.

†† 45 C.F.R. part 46.102(l)(2), 21 C.F.R. part 56; 42 U.S.C. Sect. 241(d); 5 U.S.C. Sect. 552a; 44 U.S.C. Sect. 3501 et seq.

§§ Retrospective slope 10 is a method for retrospectively characterizing RSV seasons that captures a high percentage of PCR detections. It uses a centered 5-week moving average of RSV detections normalized to a season peak of 1,000 detections. The season onset is defined as the second of 2 consecutive weeks when the slope, or normalized 5-week moving average of RSV detections between subsequent weeks, exceeds 10. The season offset is the last week when the standardized (normalized) detections exceed the standardized detections at onset.

¶¶ The Respiratory Syncytial Virus Hospitalization Surveillance Network conducts active, population-based surveillance for laboratory-confirmed RSV-associated hospitalizations (https://www.cdc.gov/rsv/research/rsv-net/dashboard.html). The New Vaccine Surveillance Network conducts active, population-based surveillance for RSV-associated acute respiratory illness among children in outpatient, emergency department, and hospital settings at seven U.S. medical centers. https://www.cdc.gov/surveillance/nvsn/index.html
